# Important aspects in the assessment of bilingual children with suspected language impairment: The Vienna Model

**DOI:** 10.1007/s40211-020-00361-x

**Published:** 2020-10-13

**Authors:** Brigitte Eisenwort, Carolin Schmid, Maksim Tilis, Dmitrij Tsoy, Gabriela Diendorfer-Radner, Anika Sedlaczek, Claudia Klier

**Affiliations:** 1 Department of Pediatrics and Adolescent Medicine, Medical University of Vienna, Währinger Gürtel 18–20, 1090 Vienna, Austria; 2 Acoustics Research Institute, Austrian Academy of Sciences, Vienna, Austria; 3 Department of Otorhinolaryngology, Division of Speech and Language Therapy, Medical University of Vienna, Vienna, Austria; 4 Comprehensive Center of Pediatrics, Medical University of Vienna, Vienna, Austria

**Keywords:** Multilingual language acquisition, German, Russian, Phonological disorder, Native speaker, Mehrsprachiger Spracherwerb, Deutsch, Russisch, Phonologische Störung, Native Speaker

## Abstract

**Background:**

Due to demographic changes over the last few decades, the number of multilingual children has grown rapidly. Many of them face problems in learning their second language. Similarities between linguistic manifestations of stages of second language acquisition and an impairment of language acquisition cause a diagnostic dilemma. The Vienna Model of language assessment in multilingual children will be presented.

**Methods:**

A key feature of our procedure is the integration of medical students as native speakers in diagnosing acquisition of the first language. A case study of a boy with Russian as first language illustrates the procedure.

**Results:**

The Vienna Model of language assessment in multilingual children offers the possibility to evaluate language competence in a differentiated manner with support of medical students as native speakers. Based on the bilingual assessment on different linguistic levels the diagnosis ICD-10 F80.0 is given. The subsequent short therapy showed an improvement regarding phonological competence.

## Abstract (de)

**Grundlagen:**

Durch die Migrationsströme der letzten Jahrzehnte wachsen immer mehr Kinder mehrsprachig auf. Viele dieser Kinder zeigen Probleme beim Erlernen der Zweitsprache. Dadurch entsteht ein diagnostisches Dilemma, da es Ähnlichkeiten zwischen den Stadien des unvollkommenen Zweitspracherwerbs und einer Sprachentwicklungsstörung gibt. Im Folgenden wird das „Wiener Modell“ der Sprachdiagnostik bei mehrsprachigem Aufwachsen vorgestellt.

**Methodik:**

Hauptmerkmal unseres Vorgehens ist die Integration von Medizinstudierenden als Native Speaker bei der muttersprachlichen Diagnostik. Anhand eines Fallbeispiels eines Buben mit Muttersprache Russisch wird das Vorgehen dargestellt.

**Ergebnisse:**

Das „Wiener Modell“ eröffnet die Möglichkeit, die sprachlichen Fähigkeiten von Kindern unter Mitarbeit von Medizinstudierenden als Native Speaker differenziert zu erfassen. Basierend auf der bilingualen Sprachdiagnostik auf mehreren sprachlichen Ebenen wird die Diagnose ICD-10 F80.0 gestellt. Eine anschließende Kurztherapie zeigt eine Verbesserung in der phonologischen Kompetenz.

## Background

Due to demographic changes over the last few decades, the number of multilingual children has rapidly grown. In Austria 45% of children in day nurseries grow up with a first language (L1) that is not German [1]. In this situation many professionals face a diagnostic dilemma because there are similarities between linguistic manifestations of stages of second language acquisition (L2) and an impairment of language acquisition [2].

About 5–8% of monolingual children show a specific language impairment (SLI) according to the criteria of ICD 10 (www.who.int/classifications/icd/en/). Even though there is not enough data available on bilingual children, it is supposed that these children suffer from SLI just as often [3].

Today testing in all languages can be seen as the gold standard. Thordadottier [4] describes four scenarios depending on the L1: (1) normed tests are available, (2) appropriate tests are not available, (3) no tests, but a clinical tradition is available and (4) neither tests nor a clinical tradition exists.

But even considering scenario 1 in which the L1 of a child can be assessed with a normed test, the high degree of possible variability of its L1 caused by the complex interaction of several factors, must be considered. Some of these factors like regional, social and situational variation are also applicable for monolingual speakers, and others are only relevant in the case of migration, caused by the changing language environment. Thus, languages inherently feature a certain, often strong, degree of variation in relation to the construct of the officially defined standard language. According to Weinreich (cited in [5, p. 218]), “*a language is a dialect with an army and a navy*” what means that only one of the language varieties is (politically) selected to be the standard language. This standard language provides the norms for the child’s language development assessed by language tests. Children speaking a variety “without an army and a navy” have the disadvantage that their receptive and expressive language abilities usually do not fall within the mean range of the expected standard language ability. Therefore, a reliable assessment of the languages of a child with migration background has to consider the multiple factors influencing the language acquisition in the framework of a successive bilingual language acquisition within an L2 environment.

As a consequence current conditions like overdiagnosis of SLI for bilingual children in many countries like Austria are based on the absence of assessment tools, the common trend to assess linguistic competence only in L2, the problem how to handle variation and the needs of the migrant families with their diverse values, beliefs and behaviors guided us to develop the following procedure.

### The Vienna Model

A core feature of our approach is the assessment of the L1 of the child together with a native speaker. In addition to the linguistic benefit, a native speaker improves the cultural competence of the assessment team because beside the challenge to assess linguistic competence in two or more languages it is important to realize that families with migration background need special care which meets their social and cultural needs [6].

### Training of native speakers

At Vienna Medical University as well as at other international (medical) universities ideal prerequisites are given for the inclusion of native speakers in the assessment of multilingual children suspected to have a language impairment. At our university about 15–20% of students have an L1 other than German. Students who are interested in supporting our counselling hour^1^ as native speakers receive an introduction, which depends on the scenario (Table 1; [4]).

**Table 1:** Languages of patients who visited our outpatient department of psychosomatics in 2017

Language family	Language
Slavic	Bosnian-Croatian-Serbian, Polish, Russian, Bulgarian
Romance	Spanish, Romanian
Northeast Caucasian	Chechen
Finno-Ugrian	Hungarian
Turkic	Turkish
Germanic	German, English
Iranic	Persian
Indoaric	Bengali
Austroasiatic	Vietnamese
Sinitic	Mandarin
Afroasiatic	Arabic
Dravidic	Malayalam

## Case study

**Fig. 1:**
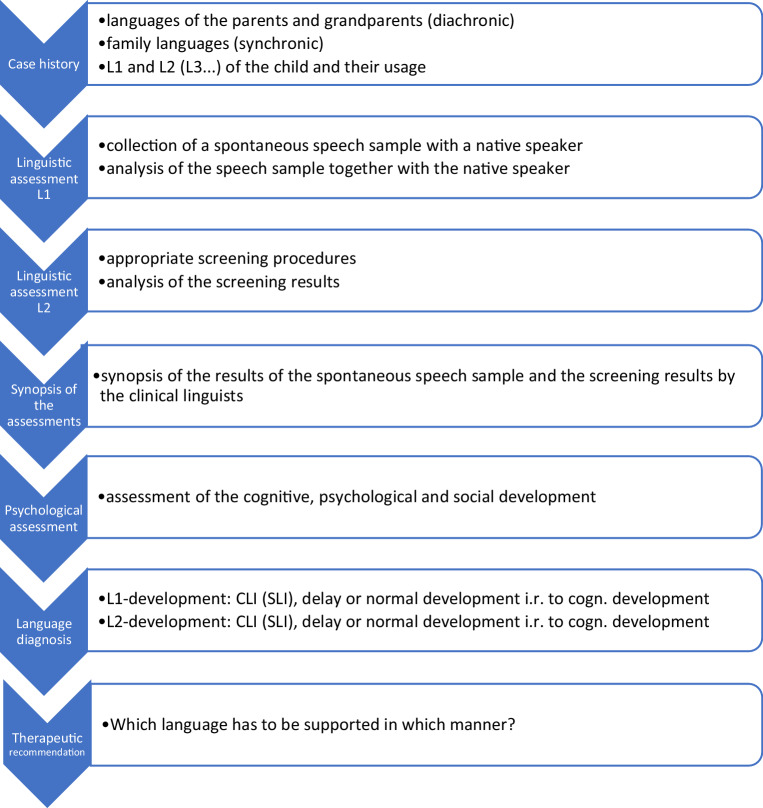
Assessment procedure. *L1* first language, *L2* second language, *L3* third language, *CLI* comorbid language impairment, *SLI* specific language impairment

Our procedure (Fig. 1, compare [7]) will be illustrated by a case study of a Russian–German bilingual boy.

### Case history

Josef (4.7 years) lives with his family and has a sister (7 years). After pregnancy and birth without any problems, Josef showed a normal babbling period and produced his first words around the time of his first birthday. He was difficult to understand for his parents, but during the last half year his intelligibility has improved. Now his parents understand by guessing about 70% of his utterances. Since 1.9 years of age he visits a private preschool, where the majority of children speak German as their L1. At home the parents speak Russian with each other and with their children. Josef and his sister speak German with each other. Josef mixes German into his Russian when communicating with his parents. While communicating with his sister he does not mix Russian into the German. German is his preferred language. The grandparents from the mother’s side acquired Surzhik, a tabooed and suppressed mixed language of Russian and Ukrainian, which does not show obvious features of reduction of linguistic complexity [8], and the L2 Russian. Josef’s mother grew up with Russian as L1. She suffered from an articulation disorder in childhood. The grandparents from the father’s side grew up with Trasjanka, a mixed language of Russian and Belorussian. Using this language does not depend on educational status, age or missing linguistic knowledge in the standard language and its linguistic structure does not show obvious signs of reduction of complexity [9]. Josef’s father also grew up with Russian as L1.

### Assessment 1

A spontaneous speech sample in the L2 German was collected and analyzed with the Percentage Consonants Correct (PCC-R) score. With a score of 52% Josef has a moderate to severe phonological disorder (Table 2; [11]).

**Table 2:** PCC-R [11]

Age before/after training (in years)	PCC-revised (in %)
4.8 before	52
5.4 after	66

*PCC-R* Percentage Consonants Correct

### Assessment 2

Hearing screening showed normal hearing. The Orofacial Praxis Test was used for evaluating the orofacial and fine motor praxis abilities. Josef scored within the mean range of the Austrian sample [12]. Language development in L2 was assessed by Lise-Daz (Table 3; [13]).

**Table 3:** Results (Lise-Daz [13])

	Percentile rank (in %)
*Comprehension*	
Verb semantics	79
Wh-questions	98
Negations	82
*Production*	
Prepositions	62
Focus particles	84
Verbs	42
Modal and auxiliary verbs	16
Conjunctions	46
4th and 3rd case	12

Except for case and modal and auxiliary verbs Josef scored in the mean range and above compared to children who acquire German as L2. Language development in L1 Russian was assessed by SRUK (Table 4; [14]).

**Table 4:** Results (SRUK [14])

Comprehension	*Nouns*	*Verbs*	*Grammatical structures*
	8/10 (normal)	8/10 (normal)	10/22 (conspicuous)
Production	*Nouns*	*Verbs*	*Case*
	16/26 (conspicuous)	11/26 (conspicuous)	1/6 (strongly conspicuous)

*SRUK* “Sprachstandstest Russisch für mehrsprachige Kinder”

Due to the fact that there are only some preliminary data for comparison instead of norms, raw scores can be classified only based on the criteria: above mean range, normal, conspicuous and strongly conspicuous [14, p. 18]. Josef scored conspicuously in many of the subtests.

### Psychological assessment

#### Cognitive ability

Josef shows a fluid intelligence above the mean range. His scores regarding spatial ability, working memory, processing speed, visual discrimination, and recall of visual information are within the mean range [15]. He also scores within the mean range in visuomotoric skills, but his scores regarding attention are beyond the mean range [16].

#### Personality

Regarding emotional problems, behavior hyperactivity and problems with peers and prosocial behavior, he is described to be without noticeable problems by his father [17].

### Diagnosis

Criteria for ICD-10, F80.0 articulation disorder (phonological disorder) are fulfilled. Language development in L2 is age appropriate, whereas Josef shows a delay in acquisition of L1. In addition to F80.0 articulation disorder (phonological disorder) without deviancies regarding orofacial and fine motor praxis abilities, he shows deficits in attention.

### Therapeutic procedure

Josef received 15 training lessons based on the training program of Fox Boyer [18]. Thereafter, 100 of Josef’s words were collected and transcribed a second time. Josef improved from a moderate–severe (50–65%) to a mild–moderate (65–85%) phonological disorder (Table 2 [11]).

## Discussion

Our central question is how results regarding linguistic skills of a child in both languages can be integrated in its cognitive, psychological and social development in the framework of a sociolinguistic background. Josef, scoring in the mean and upper mean range regarding his cognitive development, despite attention problems, is a typical bilingual boy with a stronger and a weaker language. His phonological disorder with a PCC‑R score of 52% reduces his intelligibility but nevertheless he does not show emotional problems as a consequence. De Thorne et al. find a correlation of 0.95 for articulation disorder as a hereditary disorder [10]. As mentioned above Josef’s mother also showed an articulation disorder in childhood. Regarding the diachronic sociolinguistic background, a high linguistic capital can be attested to Josef’s family. Both grandparents have spoken the standard language and a mixed language without social prestige and decided to educate their children in the standard language. In his family multilingualism occurs in diachronic and synchronic context.

Josef is a child who also could have been diagnosed with F80.0 ICD-10: articulation (phonological) disorder with a combination of language measures gathered only in his L2 and administration of a questionnaire about acquisition of L1. Nevertheless Josef as a child with weaker language skills in Russian than in German, can only be detected when both languages are assessed. Russian and German are two languages characterizing the 1st scenario [4]. Our assessment setting is characterized by an advantageous condition because of the availability of normed screening procedures in both languages as well as the opportunity to evaluate a patient, who is willing to cooperate. But for the majority of children being at risk for language impairment, the assessment setting is complicated by many factors like presence of developmental disorders, lack of motivation and compliance from the child and/or their family often due to cultural pressures, languages without normed procedures and so on. Therefore, we prefer a holistic approach in which assessment of L1 is the basis for offering more information not only for assessment of language impairment, but also for therapy planning and prognosis. Most international medical universities have a high percentage of foreign students probably interested and willing to cooperate in assessment of language-impaired children. Therefore, we also want to raise awareness on their possible contributions to support the children from their own linguistic community.

## Footnotes

1 http://kinderklinik.meduniwien.ac.at/fileadmin/kinderklinik/psychosomatik/Sprechstunde_Sprachentwicklungsst%C3%B6rungen.pdf [30 July 2020].
